# Case report: Can fibrinolytics effectively manage gross hematuria and clot retention in an elderly patients, leading to the diagnosis of lymphoma?

**DOI:** 10.1016/j.eucr.2025.103253

**Published:** 2025-10-21

**Authors:** Moein Bighamian, Maryam Sadat Jamadi, Amin Dorostkar, Farid Rajaee Rizi

**Affiliations:** a Department of Urology, Isfahan University of Medical Sciences, Isfahan, Iran; b Department of Obstetrics and Gynecology, School of Medicine, Isfahan University of Medical Sciences, Isfahan, Iran; c Isfahan Endocrine and Metabolism Research Center, Isfahan University of Medical Sciences, Isfahan, Iran

**Keywords:** Hematuria, Lymphoma, Thrombolytic therapy

## Abstract

A 77-year-old man presented with severe gross hematuria and extensive urinary tract clot retention unresponsive to catheterization and irrigation. CT showed clots throughout the upper and lower tracts; labs revealed pancytopenia and elevated creatinine. Bilateral nephrostomies with multiple Alteplase instillations dissolved clots, restored urinary flow, and normalized renal function. Bone marrow biopsy confirmed B-cell lymphoma. This case highlights fibrinolytic therapy as a minimally invasive option for clot retention and the importance of multidisciplinary evaluation in uncovering malignancies presenting with complex urologic emergencies.

## Highlights

• Alteplase successfully dissolved extensive urinary tract clots.
• Multidisciplinary approach revealed B-cell lymphoma.
• Fibrinolytics offer a minimally invasive alternative to surgery.
• Case emphasizes diagnostic vigilance in elderly hematuria.
• Restoration of renal function avoided further dialysis.

## 1 Introduction

Severe hematuria presenting with heavy clot formation is considered one of the most challenging scenarios in medical practice, especially if associated with hematologic malignancies. One such case that comes to the mind is the journey of B-cell lymphoma —a stern reminde a 77-year-old male through gross hematuria, leading eventually to the diagnosis of B-cell lymphoma—a stern reminder of the complexity involved in diagnosing and managing such cases. This patient's presentation of a rather benign-sounding urological issue, in the twilight of his life, unraveled to show a deeper and more sinister pathology, which clearly highlights the important interplay among advanced imaging techniques, interventional procedures, and multidisciplinary expertise.

Fibrinolytic agents used in the management of hematuria, especially when complicated by massive clot formation, mark a milestone in the history of urological management. Hematuria can vary from microscopic to gross and is often associated with many underlying pathologies, such as infections, trauma, malignancies, and coagulopathies. Hematuria complicated by retained clots results in obstructive uropathy, painful urination, and possible renal damage, therefore always necessitating an urgent and effective intervention.^1^^,^^2^

Fibrinolytic agents, such as Alteplase, are recombinant tissue plasminogen activators that work by converting plasminogen to plasmin, an enzyme that breaks down fibrin clots. These agents are commonly used in the treatment of thrombotic conditions, including myocardial infarction and ischemic stroke, but their application in urology, particularly for the dissolution of urinary clots, has received increasing attention in recent years.^3^

Fibrinolytic agents in such conditions of hematuria can be administered directly to the bladder or via nephrostomy tubes, especially in cases involving the upper urinary tracts. In this way, high concentrations of the agent can be achieved at the clot sites, which favors effective clot lysis with minimized systemic exposure and potential side effects.^1^^,^^4^

Clinical protocols for the use of fibrinolytics in hematuria vary, but generally include the following steps. The initial evaluation of the patient's coagulation status and renal function is essential. Imaging studies, such as ultrasound or CT scans, may be performed to determine the extent and location of clot formation. A urinary catheter or nephrostomy tube is placed to provide direct access to the site of the clot. Continuous bladder irrigation may be initiated to prevent further clot formation and maintain catheter patency. The fibrinolytic agent is instilled into the bladder or nephrostomy tube. The dosage and duration of treatment depend on the size and persistence of the clots. In some cases, repeated instillations may be necessary. Patients are closely monitored for signs of successful clot dissolution, such as improved urinary flow and reduction in hematuria. Laboratory tests and imaging studies may be repeated to assess the effectiveness of the treatment and to detect any potential complications.^1^^,^^5^^,^^6^

The use of fibrinolytic agents in the management of hematuria offers a targeted approach to clot dissolution, reducing the need for more invasive procedures such as surgical clot evacuation. However, careful patient selection and monitoring are crucial to ensure safety and efficacy. Potential risks include bleeding, infection, and allergic reactions, which must be weighed against the benefits of treatment.^5^^,^^7^

The management of hematuria, especially in elderly patients with underlying malignancies, remains a relatively underexplored area in medical research, particularly in the Middle East and Iran. While existing studies have primarily focused on the epidemiology and general treatment of hematuria, this case report introduces a novel approach by detailing the specific use of fibrinolytic agents like Alteplase for clot dissolution in complex cases. Such innovative treatment strategies, combined with a multidisciplinary approach and advanced diagnostic imaging, represent a significant advancement in the field. This case not only underscores the critical role of integrating oncology and nephrology but also highlights the importance of adopting these advanced techniques to improve patient outcomes in regions like Iran, where similar comprehensive studies are scarce.

## 2 Case presentation

A 77-year-old male presented with gross hematuria and clot retention. Initial management included the insertion of a urinary catheter and attempts to irrigate the bladder. However, the catheter became non-functional multiple times, and several attempts to evacuate the clots were unsuccessful.

**Fig. 1:**
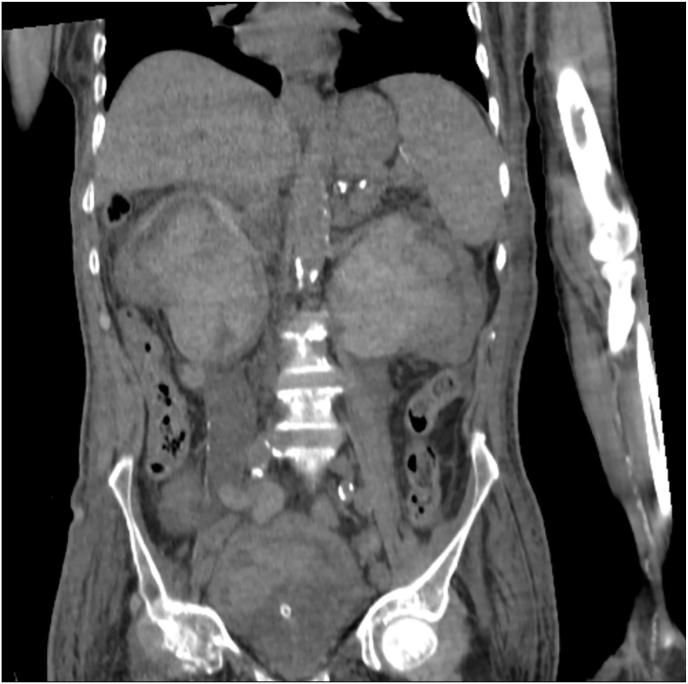
Non-contrast CT scan prior to fibrinolytic therapy, showing extensive clot burden filling the bilateral renal pelves and ureters, leading to significant hydronephrosis. The image highlights the challenging nature of the clot obstruction, extending beyond the bladder. Fig. 1 Non-contrast CT scan of the abdomen and pelvis of the patient prior to the administration of fibrinolytics.

The patient underwent a CT scan (Fig. 1), which revealed remarkable findings. The entire upper and lower urinary tracts were filled with clots, posing a significant challenge for management. Laboratory tests revealed pancytopenia and elevated creatinine levels (Cr: 3.9).

Due to the inability to irrigate the bladder effectively, bilateral nephrostomies were placed. Alteplase, a fibrinolytic agent, was administered multiple times through the nephrostomies to lyse the clots. Given the pancytopenia, a hematology-oncology consultation was obtained, and a bone marrow biopsy was recommended. The biopsy results indicated B-cell lymphoma.

**Fig. 2:**
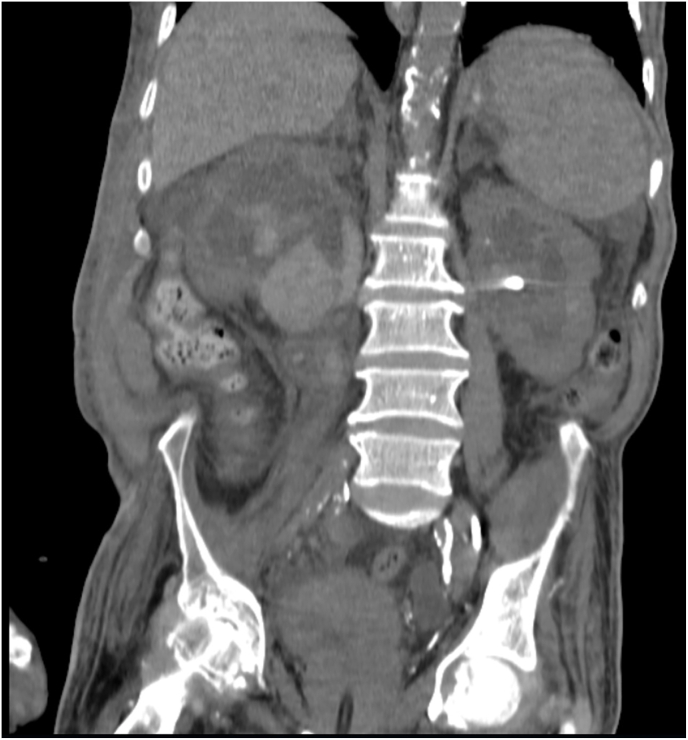
Follow-up CT scan after fibrinolytic therapy and successful clot dissolution, demonstrating marked resolution of the clot burden within the bilateral upper urinary tracts. The hydronephrosis is significantly improved, indicating restored urinary flow. Fig. 2 CT scan of the abdomen and pelvis after the administration of fibrinolytics, showing significant lysis of the clots.

During hospitalization, the patient's creatinine levels decreased and normalized to 1.2 over two weeks. Before the administration of fibrinolytic therapy, the patient underwent two sessions of dialysis based on the nephrologist's consultation due to the severity of his condition and elevated creatinine levels. However, after the fibrinolytic treatment with Alteplase, there was no further need for hemodialysis as the patient's kidney function stabilized and hematuria was effectively managed. Hematuria was controlled, and urinary output was reestablished. The follow-up CT scan (Fig. 2) showed significant resolution of the clots.

This case highlights the complexity of managing severe hematuria with extensive clot formation, especially in the context of underlying hematologic malignancy. The presence of clots throughout the urinary system complicates both diagnosis and treatment, requiring advanced imaging and interventional techniques. The discovery of B-cell lymphoma underscores the importance of a thorough diagnostic workup in patients with pancytopenia and unexplained symptoms.

Effective management of gross hematuria with clot retention in elderly patients can be challenging, particularly when complicated by conditions such as B-cell lymphoma. Advanced imaging techniques and multidisciplinary approaches are crucial for accurate diagnosis and appropriate treatment strategies. Despite successful management of hematuria and renal function, the prognosis may be poor due to underlying malignancies.

## 3 Discussion

The necessity for a multidisciplinary approach, advanced diagnostic imaging, and innovative therapeutic options is underlined in this case, as is the inclusion of fibrinolytic agents in these cases.

Hematuria can be a symptom of many diseases including infections, trauma, neoplasms, and coagulopathies. For example, the patient under consideration was finally diagnosed with B-cell lymphoma. In some instances, the prognosis worsens, and hematuria may worsen to clot retention which can result in urethral obstruction, decreased kidney function, and subsequently, an increase in morbidity, which necessitates a comprehensive diagnostic investigation, and management of the problem. In such cases, for example, CT scans are deemed necessary to determine the size of the blood clot and to confirm the clot as the underlying factor. The CT scan showed that there were blood clots in both the ureters and the kidneys, making it imperative to intervene with quick and effective management approaches 2.

The use of fibrinolytic agents like Alteplase has proven effective and has gained increasing recognition in the management of hematuria, having evolved from its initial use in thrombotic conditions. These agents activate the breakdown of blood clots by the plasminogen-to-plasmin conversion mechanism. Consequently, fibrin clots are degraded. The localization of Alteplase through the nephrostomy tubes in the patient enabled him to have a high concentration at the clot site, thus providing a highly selective and focused treatment approach to clot lysis and reducing systemic exposure and its associated risks1.

The course of fibrinolytic therapy was successful partly due to careful planning at the patient level, steering clear of inappropriate candidates, and rigorously adhering to protocols. Preliminary examination of the patient's coagulation status and renal function, together with proper imaging, was the most important step in the identification of clot and administration of fibrinolytic agents. In addition, constant bladder irrigation removed fragmented clots as well as maintained the catheter patency, thus decreasing the chance of recurrent obstructions and infections.

Although fibrinolytic therapy is a well-known treatment option in patients with hematuria, potential complications such as bleeding, infection, and associated adverse reactions should be vigilantly monitored to prevent complications. The pancytopenia in this patient underscored the need for haemato-oncology consults and biopsy that ultimately revealed a diagnosis of B-cell lymphoma. This holistic treatment approach helped keep both the presenting condition and underlying conditions optimally managed albeit the patient's prognosis was dismal as a result of the aggressive nature of the lymphoma.

The introduction of fibrinolytic agents into urological practice marks a significant milestone, offering less invasive routes to address severe hematuria compared to traditional surgical approaches. These agents, by ensuring quicker clot resolution, not only alleviate symptoms more rapidly but also reduce the risks associated with prolonged clot retention and recurrent interventions. The efficiency of Alteplase in this case, for example, underscores its potential as a cornerstone in the management of hematuria complicated by clot retention.

However, there remains a need for standardized protocols to optimize the use of fibrinolytic agents in urological settings. Variability in dosage, administration routes, and patient monitoring can affect outcomes, underscoring the necessity for well-structured clinical guidelines. Collaborations between urologists, hematologists, and radiologists can foster the development of these protocols, ensuring a balanced approach to risk and benefit.

Furthermore, the role of fibrinolytics in the context of cancer-induced coagulopathies, such as those seen in this case, warrants deeper exploration. The interplay between malignancy, clot formation, and systemic therapy calls for tailored strategies that address both symptomatic relief and underlying oncologic treatment. Future studies focusing on the combined use of fibrinolytic therapy with cancer-specific treatments can illuminate paths to improved patient outcomes, potentially transforming standards of care in these complex cases.

## 4 Conclusion

Fibrinolytic therapy represents a valuable tool in the management of hematuria with clot retention, providing a minimally invasive option for effective clot dissolution. Ongoing research and clinical experience will continue to refine the protocols and expand the indications for this innovative treatment approach.

## 5 Limitations

The case report is limited by its retrospective nature and the focus on a single patient. Further studies with larger patient populations are needed to validate the effectiveness and safety of fibrinolytic therapy in similar clinical scenarios.

## 6 Future research

This case highlights the need for further research into the optimal use of fibrinolytic agents in urology. Prospective studies and clinical trials could provide more robust evidence to guide clinical practice and improve patient outcomes.

## CRediT authorship contribution statement

**Moein Bighamian:** Writing – review & editing, Writing – original draft, Supervision, Formal analysis, Data curation, Conceptualization. **Maryam Sadat Jamadi:** Writing – review & editing, Writing – original draft, Methodology, Investigation, Data curation, Conceptualization. **Amin Dorostkar:** Writing – review & editing, Writing – original draft, Visualization, Supervision, Software, Data curation. **Farid Rajaee Rizi:** Writing – review & editing, Writing – original draft, Visualization, Validation, Supervision, Software, Resources, Project administration, Methodology, Investigation, Funding acquisition.

## Patient consent

Informed consent was obtained from the patient (or the patient's family) for all diagnostic and therapeutic procedures. Additionally, consent was obtained for the publication of this case report, ensuring that the patient's privacy and confidentiality were maintained.

## Ethics

The case report was conducted in accordance with the ethical standards of the institution and the Declaration of Helsinki.

## Ethics approval and consent to participate

Written informed consent was obtained from the patient's parent for publication of this case report and accompanying images.

## Use of artificial intelligence

Artificial intelligence (AI) was used solely for text refinement and English language editing during manuscript preparation. All scientific content, analysis, and clinical interpretation were provided by the authors.

## Declaration of generative AI in scientific writing

During the preparation of this work, the authors used ChatGPT Enterprise was used solely for text refinement and language improvement. The authors reviewed and edited the content as needed and take full responsibility for its content. The AI tool was used solely for linguistic improvement and did not contribute to the conceptualization, data interpretation, or clinical decision-making presented in this report.

## Funding

This report received no external funding from public, commercial, or not-for-profit sources.

## Conflict of interest

The authors declare no conflict of interest. The use of Alteplase and other treatments was based on clinical judgment and standard medical practice, without any influence from pharmaceutical companies or other external entities.
